# Direct and multiplex quantification of protein biomarkers in serum samples using an immuno-magnetic platform†
†Electronic supplementary information (ESI) available. See DOI: 10.1039/c5sc04115e


**DOI:** 10.1039/c5sc04115e

**Published:** 2016-01-04

**Authors:** See-Lok Ho, Di Xu, Man Shing Wong, Hung-Wing Li

**Affiliations:** a Department of Chemistry , Hong Kong Baptist University , Hong Kong . Email: mswong@hkbu.edu.hk ; Email: hwli@hkbu.edu.hk

## Abstract

A direct and ultrasensitive multiplex assay using an immuno-magnetic platform has been developed for the quantification of trace amounts of circulating cancer-associated antigens in serum. The detection is based on the specific immuno-interactions among the target antigen, detection antibody and capture antibody that is immobilized on the surface of magnetic nanoparticles. The sandwiched immuno-assembly is then labelled with turn-on fluorophores and detected with a fluorescence imaging system. To afford a high signal-to-noise ratio, three turn-on fluorophores with unique optical properties have been designed and synthesized to label the target antigens. The developed assay has achieved a remarkable LOD down to the femto-molar regime without sample pre-treatment. This versatile assay can efficiently differentiate the target antigen from a protein matrix and simultaneously quantify multiple cancer-associated antigens, for instance, alpha-fetoprotein (AFP), carcinoembryonic antigen (CEA), and prostate specific antigen (PSA) using only 6 μL of serum sample in an hour. This novel system has a high applicability to serve as a universal and useful tool for early disease diagnostics.

## Abstract 2

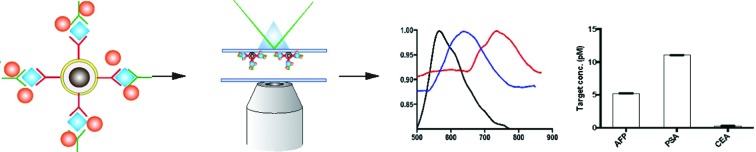
A direct and ultrasensitive multiplex assay using an immuno-magnetic platform has been developed for the quantification of trace amounts of circulating cancer-associated antigens in serum.

## Introduction

Over the past decade, protein biomarkers have attracted great interest in early disease diagnosis, including cancers, inflammation and neurodegenerative diseases.^1^–^3^ As circulating protein biomarkers such as cancer specific antigens are found in a wide range of body fluids (liquid biopsy) and the changes in their expression profile is highly correlated to the occurrence and malignancy of a particular cancer;^4^–^7^ these biomarkers are considered as a non-invasive tool for early cancer diagnosis.^8^,^9^ Cancer development, for instance, is a multistage process that involves different biomarkers. The simultaneous detection of multiple biomarkers and establishment of an expression profile can therefore certainly enhance the diagnostic accuracy and facilitate early clinical treatment for a higher survival rate.^10^,^11^ Unfortunately, the current standard methods, such as ELISA and immuno-blotting,^12^,^13^ often require cumbersome multiple purification and pre-treatment processes, and more importantly, these assays are at best only semi-quantitative. Furthermore, to quantify the trace amount of biomarkers in the complex sample matrix such as serum, these standard methods often require a large amount of sample. These problems often hinder the accuracy and throughput of the assays and therefore limit their clinical applications. In contrast, a simple, direct and yet sensitive and specific detection assay will advance early cancer diagnosis to the next level.

Herein, we have developed an assay for specific multiplex detection of disease-related antigens with three newly developed turn-on cyanine fluorophores, namely, **SLAce**, **SPAce**, and **VLAce**. Cyanine has been reported to be a useful and highly sensitive fluorescence probe that exhibits high binding affinity towards double-stranded DNA and beta-amyloid species.^14^,^15^ In contrast to the commercially available protein labeling dyes, such as FITC, Cy3 and Cy5, these cyanine fluorophores show a strong fluorescence enhancement (>80-fold) upon binding to the biomolecules, enabling the highly sensitive detection of biomarkers. Moreover, the three newly developed dyes can be excited at 488 nm but emit distinctively different wavelengths, which facilitate the simultaneous detection of multiple target biomarkers under a single excitation light source. Most importantly, the preparation of the cyanine compounds is relatively simple and inexpensive when compared to other organic protein labeling dyes.

For detection, the target analytes are captured and detected on the surface of the nanoparticles based on the specific immuno-interactions between the target antigens and the specific antibodies. Magnetic nanoparticles (MNP) are utilized as the pre-concentration platform due to the fact that the magnetic particles can pull the conjugated biomolecules from a laminar path to another upon applying an external magnetic field and therefore selectively separate the target analytes from the sample matrix without additional offline purification and washing steps.^16^–^18^ Therefore, the magnetic nanoparticle conjugated sandwich immunoassay developed herein offers the advantages of sandwich immunoassays, which include the high specificity and binding affinity of the antibody and antigen. In addition, the utility of magnetic nanoparticles allows online pre-concentration and purification and therefore results in a direct, simple and accurate detection method. As a demonstration, three different cancer-associated antigens, alpha-fetoprotein (AFP), carcinoembryonic antigen (CEA), and prostate specific antigen (PSA) were chosen as the target analytes. AFP is a biomarker that is well-known to be associated with hepatocellular carcinoma and other malignancies,^19^,^20^ whereas abnormal concentrations of CEA and PSA in serum appears to be a sign of colorectal carcinoma^21^ and prostate cancer,^22^ respectively. The assay was verified to be capable of quantifying the antigens in a serum sample and the results were further validated using ELISA.

## Results and discussion

### Design of the labelling fluorophore

**Fig. 1:**
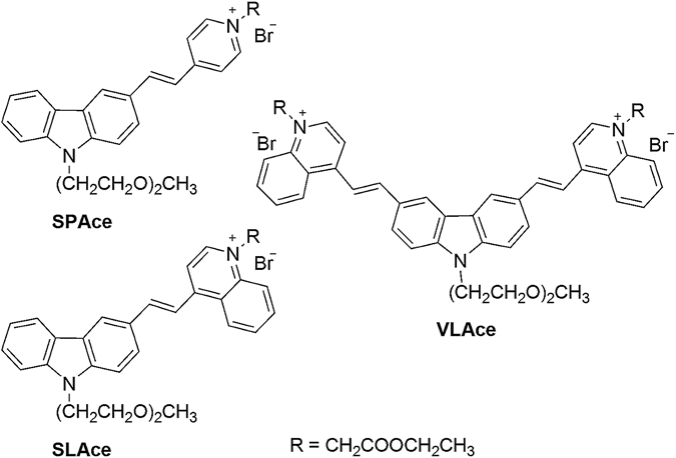
The molecular structures of the cyanine fluorophores.

The molecular structures of the tailor-made cyanine fluorophores are shown in Fig. 1. The syntheses of **SLAce**, **SPAce**, and **VLAce** are outlined in Scheme S1 of the ESI.† The cyanine skeleton was synthesized either using a Knoevenagel condensation or palladium-catalyzed Heck coupling as the key step. The proposed structures were fully characterized by ^1^H NMR, ^13^C NMR, and HRMS, which are in good agreement with the spectral data reported in the literature. The photophysical properties of these three new cyanines measured in PB solution are summarized in Table S1.† They mainly exhibited a very strong and broad charge-transfer absorption band with an absorption maximum in the range of 429–524 nm in PB solution. Upon excitation at 488 nm, these cyanines exhibited distinct fluorescence with different emission maxima ranging from 588 to 723 nm. Because of the fast non-radiative decay caused by the strong and dynamic adhesive interactions with water molecules, the fluorescence quantum yields of these cyanines are often low (<0.1) in PB solution in contrast to the moderate fluorescence obtained in an organic solvent such as DMSO. In addition, upon binding with a host, a strong increase in fluorescence will result giving rise to a turn-on fluorescence mechanism that can be used for the detection of a host. For instance, upon mixing with bovine serum albumin (BSA), there is a strong increase in the fluorescence intensity of 7–16 folds for these cyanines. We anticipated that the interaction between proteins and cyanine fluorophores was mainly hydrophobic and π–π stacking interactions as suggested by the blue-shift in the emission peak of the fluorophores upon binding to the proteins.

### Detection strategy

**Fig. 2:**
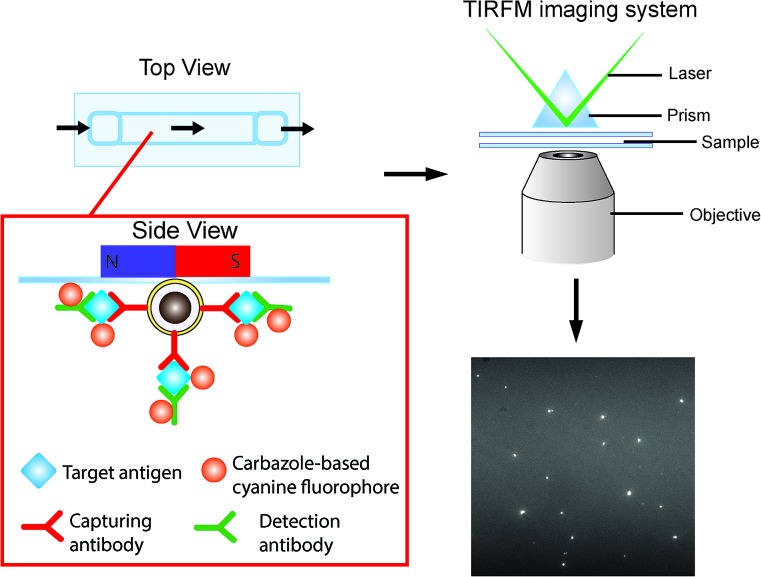
Schematic of the direct detection of the single sandwiched immuno-assembly with total internal reflection fluorescence microscopy (TIRFM). Silica was coated on the surface of the iron oxide particles *via* base-catalyzed hydrolysis of tetraethyl orthosilicate (TEOS) in a layer-by-layer assembly fashion. Subsequently, the surface of the nanoparticles was further modified with APTES and conjugation of the capture antibody was carried out using a cross-linker, glutaraldehyde (GA). The target antigen and detection antibodies were added. The sandwiched magnetic immune-assembly (MIA) was labeled using a turn-on cyanine fluorophore. The fluorescence signal of each MIA was recorded and analyzed.

The detection scheme is illustrated in Fig. 2. Upon the formation of the sandwich immuno-assembly among the capture antibody (Ab1), target antigen, and the detection antibody (Ab2) on the surface of the MNP, the magnetic immuno-assembly (MIA) will be labeled with the tailor-made cyanine fluorophores. The fluorescent MIAs were then introduced into a homemade glass flow cell; the MIAs were then separated from the bulk solution and immobilized on the top coverslip by an external magnetic field. Then, the fluorescent MIAs were visualized and detected under a TIRFM-EMCCD imaging system using a 488 nm cyan excitation laser for quantitative analysis. As the evanescent field generated by the total internal reflection was very shallow, only analytes that are located within the evanescent field are excited by the laser (in this case the MIAs that are immobilized at the top coverslip/water interface), whereas the rest in the bulk solution remains silent. The TIRFM improves high signal-to-noise images of the MIAs.

Silica-coated iron oxide nanoparticles were prepared. The TEM (Fig. S1†) images revealed that the particles were round in shape with an average diameter of 162 ± 25 nm, an average silica shell thickness of 55 ± 6 nm and bare iron oxide nanoparticles of average diameter 5.3 ± 1.0 nm. The as-prepared MNP dispersed in solution was readily attracted by a small magnet.

For the optimal performance of the detection assay, we firstly studied the concentration dependence of the capture antibody that was applied to immobilize on surface of the MNP. As shown in Fig. S2A,† the coverage of capture antibody (Ab1) was saturated at an applied concentration of 1 nM as suggested by the highest fluorescence signal obtained from the cyanine, **SLAce**, labeled antibodies conjugated MNPs. Therefore, Ab1 at a concentration of 1 nM was applied throughout the entire study. Although the higher population of the MNP immobilized on the flow cell for detection increases the throughput of the assay, the target antigens might distribute themselves among the MNPs and consequently, a “diluted” signal may be generated. An optimal density of the magnetic nanoparticles can improve the sensitivity of the assay. To study the optimal amount of the MNPs to be applied in the assay, one of the cyanine fluorophores, **SLAce** and PSA were initially chosen as the labeling dye and the target antigen, respectively. As shown in Fig. S2B,† the fluorescence signal detected with 20 mg mL^–1^ nanoparticles was much lower than 10 mg mL^–1^ and 5 mg mL^–1^, which agreed with our prediction that a high population of the nanoparticles will weaken the signal generated by the immune-assembly. In addition, the fluorescence signal tends to decrease when the amount of nanoparticles was diluted to 5 mg mL^–1^. The low density of the nanoparticles might limit the chance of the target reaching the detection platform and therefore fewer targets will be captured on the nanoparticles. To obtain a sensitive detection of the target PSA, 10 mg mL^–1^ of the magnetic nanoparticles was applied for the rest of the experiments. To further optimize the performance of the system with respect to the applied concentration of the labeling fluorophores, as shown in Fig. S2C,† the net intensity of single MIA was highly correlated to the final concentration of the dye from 10 nM to 100 μM. Beyond 100 μM, the increasing amount of dye was expected to enhance the signal generated, but the background fluorescence of the MIA probe was also increased and resulted in a decreased net intensity. The result suggested that 100 μM was the optimum dye concentration for detection. The stability of the dyes against salt and photo-irradiation was assessed and is shown in Fig. S3† (stability against photo-irradiation) and Fig. S4† (stability against salt, NaCl). The three cyanines are generally photo-stable under excitation at 488 nm. However, the fluorescence signals of the dye-labeled proteins are salt concentration dependent. The higher the salt concentration added, the lower the fluorescence intensity. The presence of salt would govern the dye–protein interactions and the solubility of the cyanine dye in the buffer solution. The effect of the magnetic nanoparticles on the fluorescence of the protein–dye complexes has been investigated and is shown in Fig S5.† The magnetic nanoparticles did not influence the resultant fluorescence signal of the dye labeled proteins.

### Standard curve

**Fig. 3:**
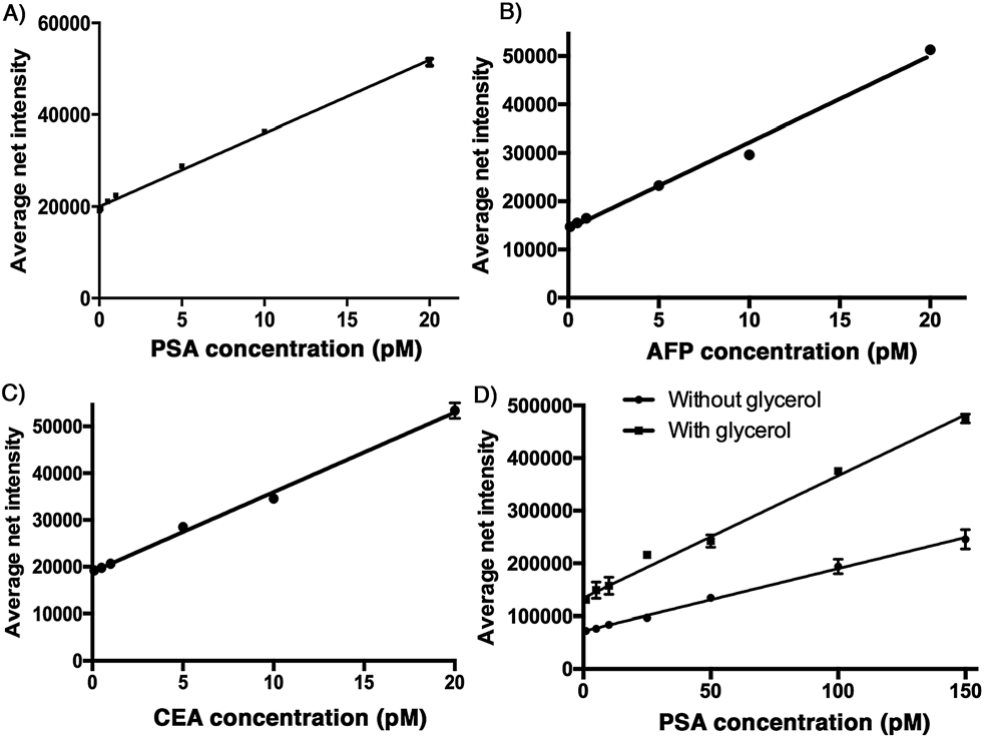
Calibration plots for the quantification of A) PSA, B) AFP, and C) CEA. Different concentration of antigens were incubated with the Ab1-MNP and the Ab2, error bar, standard error of mean, *n* = 3. D) Calibration plot for the quantification of PSA in the presence of 10 % glycerol (top) and in the absence of 10 % glycerol (bottom), error bar, standard error of mean, *n* = 3. (Average net intensity = (1×1 sq pixel of 50 individual MICs) – (1×1 sq pixel of 50 individual background area on the image)/50)

To demonstrate the sensitivity of the developed assay, a calibration plot of the average net intensity as a function of the concentration of the target antigen was constructed under the optimal conditions mentioned above. Briefly, a target antigen of concentration ranging from 0 to 20 pM was incubated with 10 mg mL^–1^ Ab1-MNPs and 100 pM Ab2 at 37 °C. The resultant MIAs were then labeled with 100 μM **SLAce** (PSA), **SPAce** (AFP), and **VLAce** (CEA). By measuring the fluorescence intensity of 50 individual MIAs as a function of the target antigen concentration, the calibration curves (Fig. 3A–C) were achieved with a good linear correlation coefficient with *R*^2^ = 0.996, *R*^2^ = 0.991 and *R*^2^ = 0.996 for SLAce labelled PSA composites, **SPAce** labelled AFP composites and **VLAce** labelled CEA composites, respectively. A limit of detection (LOD) for PSA 200 fM (6.5 pg mL^–1^) (LOD = blank + 3 × standard error of mean of blank) and a limit of quantification of 2 pM (0.66 ng mL^–1^) (LOQ = blank + 10 × standard error of mean of blank) were obtained. Moreover, the LOD for AFP and CEA was determined to be 300 fM and 600 fM, respectively. With reference to the typically recognized serum cut-off value of PSA, AFP and CEA set at 4 ng mL^–1^,^23^,^24^ 6 ng mL^–1^ (87 pM)^25^ and 2.5 ng mL^–1^ (13.8 pM),^26^ respectively, this detection assay was able to quantify the trace amounts of cancer associated antigens in serum samples for clinical application. To further enhance the fluorescence signal given by the cyanine fluorophore, 10% of glycerol was added to the MIAs as studies have shown that an increase in solvent viscosity will hinder the rotation of the molecular rotor and therefore restrict the quenching pathway of the cyanine fluorophore leading to the fluorescence enhancement.^27^ To demonstrate this phenomenon two calibration curves for the detection of PSA: with and without glycerol were constructed. As shown in Fig. 3D, the addition of glycerol almost doubled the fluorescence signal generated from the **SLAce** conjugated MIA. Although the addition of glycerol also enhanced the background fluorescence signal (probe-only) and therefore unable to significantly improve the detection limit, the presence of glycerol can further enhance the sensitivity of the overall detection assay (the slope in Fig. 3D), suggesting that the addition of glycerol can facilitate the detection when a less sensitive detector such as a conventional spectrofluorometer was used. To demonstrate the capability of point-of-care detection with a conventional fluorometer, a calibration curve of the fluorescence intensity measured at 654 nm (emission wavelength of **SLAce** upon binding to protein) as a function of the PSA concentration was constructed under the optimal conditions with 10% glycerol. As shown in Fig. S6,† the assay has achieved a linear range from 0 to 200 pM of PSA with a good linear correlation coefficient, *R*^2^ = 0.962 and a LOD of 93 pM, which was below the serum cut-off value of PSA. This confirmed the feasibility of the assay for point-of-care detection with a conventional fluorometer. The assay for the detection of protein biomarkers with TIRFM imaging system can provide a sensitive quantification using trace amounts of sample, whereas the detection with a commercial fluorometer can facilitate a rapid and simple screening for protein biomarkers.

### Selectivity of the assay

**Fig. 4:**
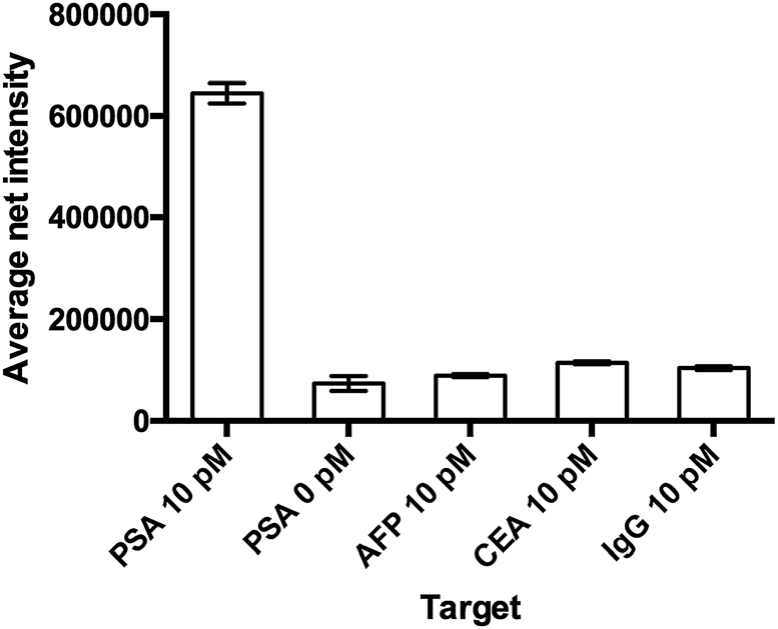
The study of the selectivity of the assay. The probe was capable of differentiating the target from other proteins. Error bar, standard error of mean, *n* = 3. (Average net intensity = (1 × 1 sq pixel of 50 individual MIAs) – (1 × 1 sq pixel of 50 individual background area on the image)/50).

The specificity of the detection probe plays an important role in the accuracy and sensitivity of a detection assay. To demonstrate the selectivity of the developed assay, four different human antigens and protein samples (AFP, CEA, PSA and IgG) with a final concentration of 10 pM were added and incubated with 10 mg mL^–1^ Ab1 conjugated nanoparticles and 100 pM Ab2 under the optimal conditions and labeled with 100 μM **SLAce**. IgG is the major component and the most abundant antibody isotype in human serum.^28^,^29^ The fluorescent images of the MIAs were captured using the TIRFM imaging system. As illustrated in Fig. 4, the false hit rate in the samples containing AFP, CEA and IgG was only 2.4%, 6.3% and 4.7%, respectively ((signal from sample – blank)/(signal from PSA – blank) × 100%). This indicated that the Ab1 conjugated nanoparticles and the Ab2 have a high binding affinity towards the target and are capable of selectively discriminating the target antigen from the others.

### Detection of PSA in a serum sample

**Fig. 5:**
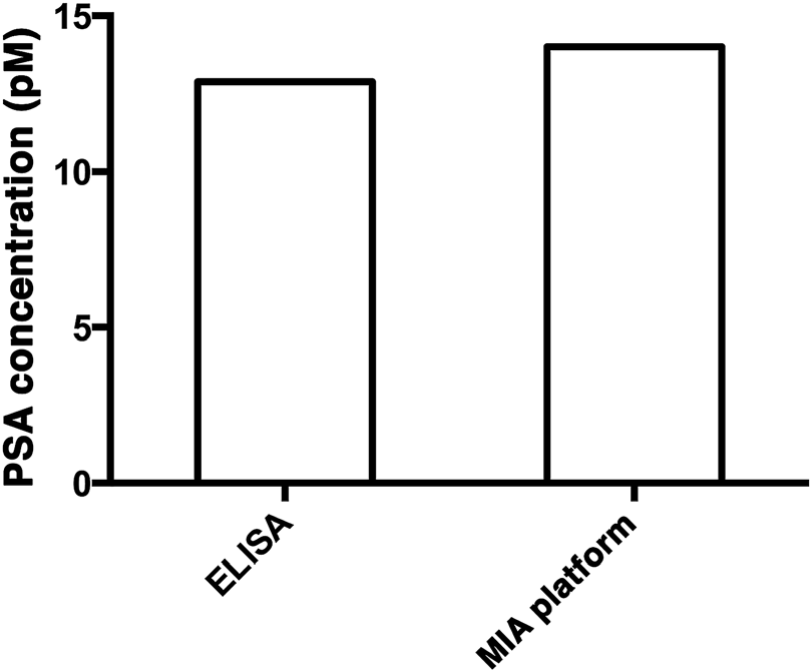
The quantification of PSA in a crude serum sample using a commercially available PSA ELISA kit (left) and the MIA assay (right).

To demonstrate the capability of quantifying the trace amount of antigen in serum sample, the developed system was applied to detect the PSA level in a crude serum sample of a healthy young male donor. An external calibration curve (Fig. 3A) was constructed for the determination of the serum PSA concentration. As illustrated in Fig. 5, the concentration of PSA in the serum sample was found to be 14 pM. The result agreed very well with that obtained using a commercially available ELISA kit, which yielded 12.9 pM and was far below the serum cut-off point for prostate cancer (122 pM). The developed assay thus provides a novel option for early cancer diagnosis.

### The simultaneous detection of cancer specific antigens in a serum sample

**Fig. 6:**
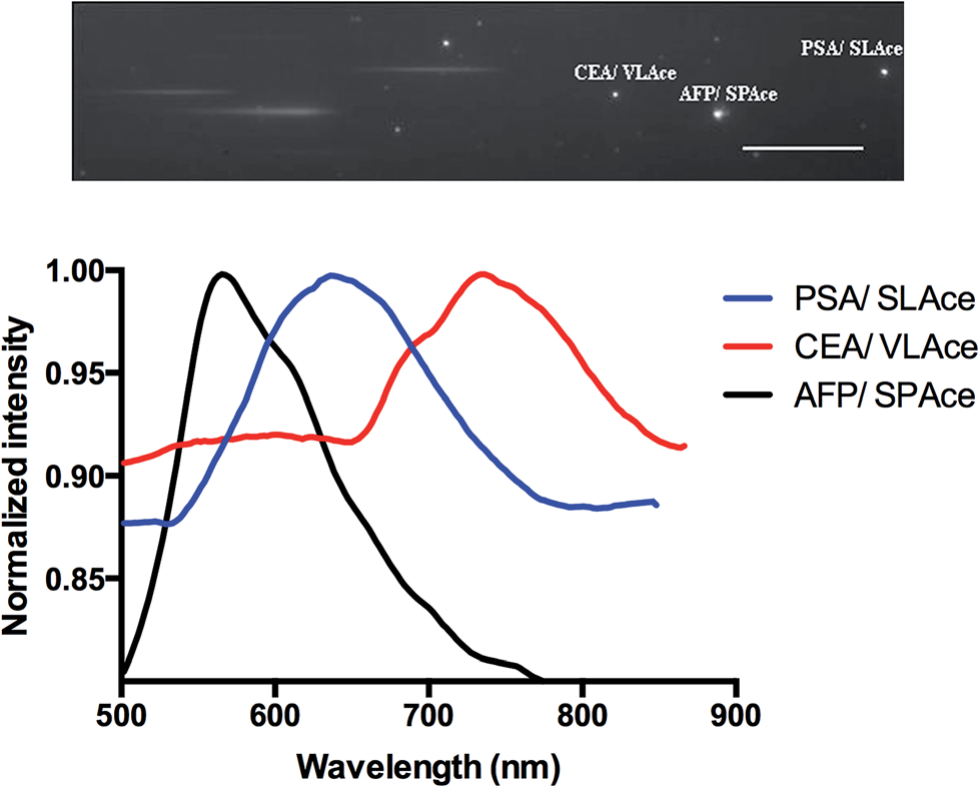
The multiplex detection of cancer associated antigens: zero and first order images of the immune-assemblies labeled with **SPAce**, **SLAce** and **VLAce** (top), and the corresponding emission spectra of the magnetic immune-assemblies (bottom).

To study the multiplexity of the detection assay, 10 pM of the target antigens, AFP, CEA, and PSA, were incubated in solution with the corresponding Ab1-MNP probe and Ab2. Then, the MIAs were further labeled with the tailor-made cyanine fluorophores, **SPAce**, **VLAce**, and **SLAce**. The solution mixture of the MIAs was then injected into the flow cell. The first-order fluorescent images were visualized under the TIRFM-EMCCD imaging system coupled with a transmission grating. As shown in Fig. 6, the **SPAce**-labeled AFP MIAs, **VLAce**-labeled CEA MIAs, and **SLAce**-labeled PSA MIAs exhibited emission peaks at 570 nm, 750 nm, and 650 nm, respectively. The emission peaks matched with the emission bands of the corresponding cyanine fluorophores upon binding to PSA MIAs, as shown in Fig. S7.† From the resolved spectra obtained from the first order images, the individual MIAs were recognized and differentiated from one and other. The quantification of each of the target antigens was also achieved by simply measuring the fluorescence signal of the corresponding MIAs.

**Fig. 7:**
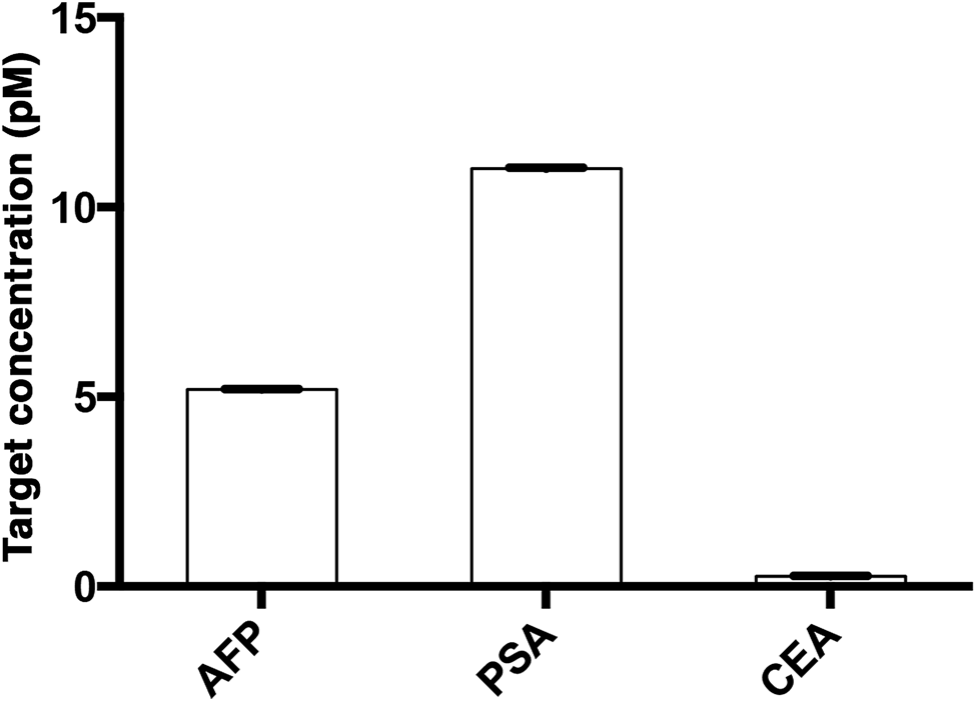
The multiplex quantification of the cancer-associated antigens AFP, PSA, and CEA labeled with **SPAce**, **SLAce** and **VLAce**, respectively.

The assay was further applied to simultaneously quantify the three cancer biomarkers in a serum sample. The MIAs capturing AFP, PSA and CEA were labeled with **SPAce**, **SLAce**, and **VLAce**, respectively. The concentration of the captured antigen from the sample was determined by measuring the peak intensity of the resolved spectra from the first-order image of the nanocomposites. A standard curve was constructed as a reference (Fig. S8†). As shown in Fig. 7, the expression profile for AFP, PSA, and CEA in the crude serum was readily found to be at a concentration of 5.2 ± 0.014, 11 ± 0.023 and 0.27 ± 0.005 pM, respectively. As the concentration of AFP and CEA detected was below the serum cut-off values, the 6 ng mL^–1^ (87 pM)^25^ and 2.5 ng mL^–1^ (13.8 pM)^26^ and PSA concentration agreed with the ELISA values and all the results are below the cut-off point of the corresponding cancer,^30^–^33^ the system was demonstrated to be capable of simultaneously detecting the three disease related antigens in 6 μL of a crude serum sample. We anticipated that any other protein biomarkers could be determined in the same manner for a more intensive expression profile and accurate disease diagnostics and prognostics.

## Conclusions

In summary, a simple, direct, sensitive and specific assay for the multiplex detection of disease-associated antigens using an antibody bio-conjugated magnetic probe and tailor-made turn-on fluorophores with TIRFM imaging system has been developed. The newly developed system is capable of selectively differentiating the target antigens from other proteins in the matrix and can achieve a remarkable LOD down to the femto-molar regime. This assay was further applied to quantify the amount of target cancer antigens in a serum sample without sample enrichment and the results obtained agreed well with those obtained using a commercially available ELISA kit. The developed assay has great potential to serve as an analytical tool for early disease diagnosis, progression monitoring and staging. Furthermore, this approach is universal and the as-developed assay can be readily modified and elaborated further, such as replacing the antibodies with other disease-associated antibodies, nucleic acid probes, or aptamers for a broad range of biomedical research and disease diagnostics.

## Supplementary Material

Supplementary information
