# Supplementary material for: Direct and multiplex quantification of protein biomarkers in serum samples using an immuno-magnetic platform
Source: Chem Sci. 2016 Jan 4;7(4):2695–700. doi: 10.1039/c5sc04115e (PMC5477028; doi:10.1039/c5sc04115e)
Supplement: Supplementary file 1 [file SC-007-C5SC04115E-s001.pdf]

Electronic Supplementary Material (ESI) for Chemical Science.
This journal is © The Royal Society of Chemistry 2016

Electronic Supplementary Information (ESI) for Chemical Science

Direct and Multiplex Quantification of Protein Biomarkers in Serum Sample with Immuno-magnetic Platform

See-Lok Ho[a,b], Di Xu[a,b], Man Shing Wong[a,*], Hung-Wing Li[a,*]

[a]Department of Chemistry, Hong Kong Baptist University Kowloon Tong, Hong Kong, SAR, China

[b]Contributed equally to this work

E-mail: mswong@hkbu.edu.hk; hwli@hkbu.edu.hk

Materials and Methods

Synthesis and NMR characterization of cyanine fluorophores

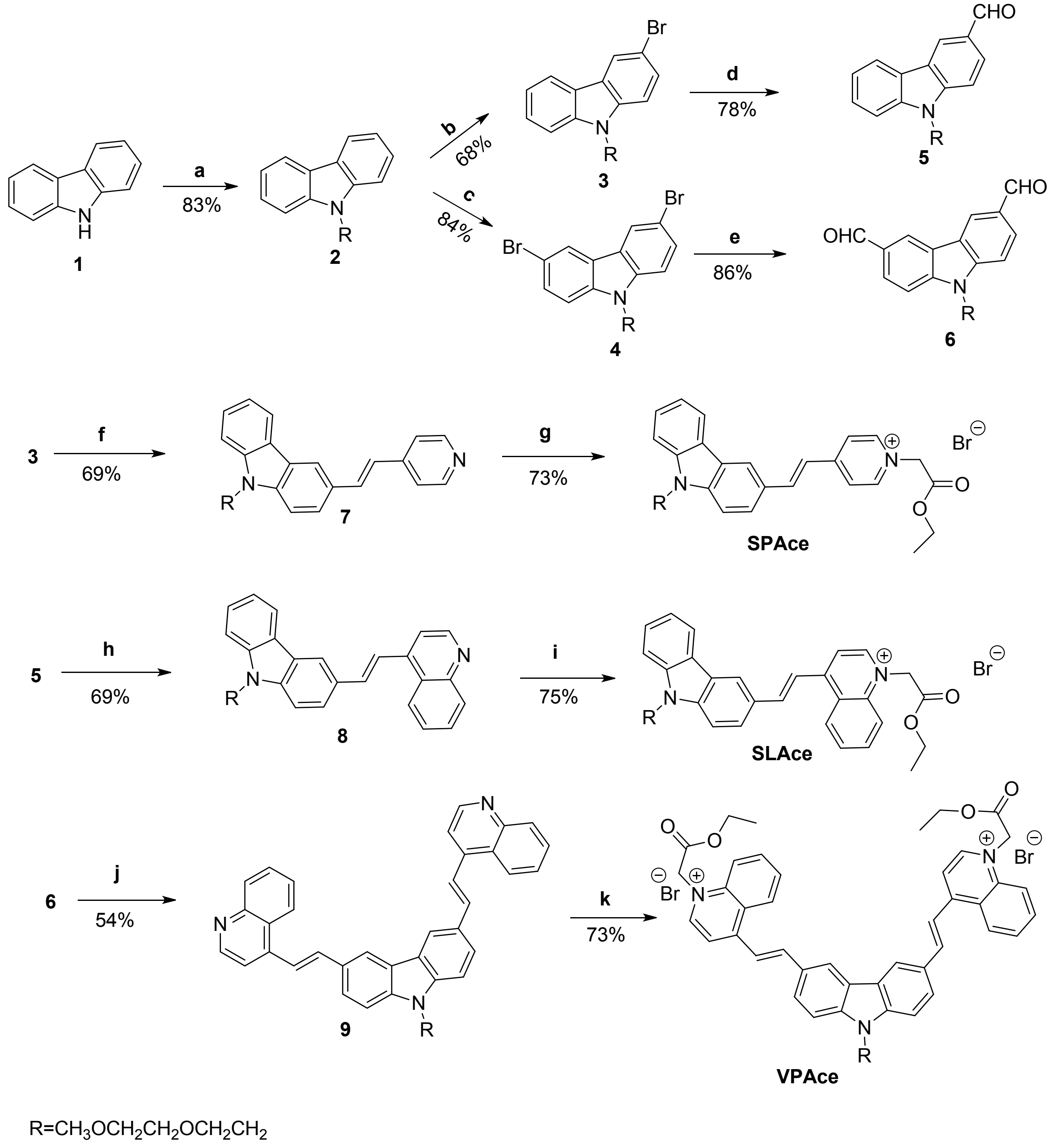

Reagents and Conditions: a, $ClCH_2CH_2OCH_2CH_2OCH_3$, NaH, DMF, 75 °C; b, N-bromosuccinimide, CH_2Cl_2, 0 °C to room temp.; c, N-bromosuccinimide, CH_2Cl_2, 0 °C; d, *n*-BuLi, DMF, THF, -78 °C; e, *n*-BuLi, DMF, THF, -78 °C; f, 4-vinylpyridine, $Pd(OAc)_2$, $P(o\text{-tol})_3$, Et3N, DMF, 90 °C; g, MeCN, $BrCH_2COOCH_2CH_3$, reflux.; h, Lepidine, chlorotrimethylsilane, DMF, 100 °C, sealed tube; i, MeCN,

$BrCH_2COOCH_2CH_3$, reflux.; j, Lepidine, chlorotrimethylsilane, DMF, 100 °C, sealed tube; k, MeCN, $BrCH_2COOCH_2CH_3$, reflux.

Scheme S1. Synthesis of cyanine fluorophores.

General Procedure All the solvents were dried by the standard methods whenever needed. ^{1}H NMR spectra were recorded using a Bruker-400 NMR spectrometer and referenced to the residue $CDCl_3$ 7.26 ppm or DMSO-d_6 2.5 ppm. ^{13}C NMR spectra were recorded using a Bruker-400 NMR spectrometer and reference to the $CDCl_3$ 77 ppm or DMSO-d_6 39.5 ppm. Mass Spectroscopy (MS) measurements were carried out by using either fast atom bombardment on the API ASTER Pulser I Hybrid Mass Spectrometer or matrix-assisted laser desorption ionization-time-of-flight (MALDI-TOF) technique.

Compound **1, 2, 3, 4, 5, 6** and **7** were synthesized according to previously published procedures.[1-3]

(*E*)-1-(2-ethoxy-2-oxoethyl)-4-(2-(9-(2-(2-methoxyethoxy)ethyl)-9H-carbazol-3-yl)vinyl) pyridine-1-ium bromide (SPAce). A solution of **7** (0.19 g, 0.5 mmol) and ethyl 2-bromoacetate (0.33 g, 2.0 mmol) in ethanol was stirred overnight at room temperature. After removing the solvent, the residue was precipitated from methanol and ethyl acetate affording **SPAce** in 73% yield. ^{1}H NMR (400 MHz, DMSO-d_6) δ 8.84 (d, J = 6.8 Hz, 2H), 8.61 (s, 1H), 8.30-8.26 (m, 3H), 8.20 (d, J = 7.6 Hz, 1H), 7.90 (dd, J = 1.6 Hz, J = 8.8 Hz, 1H), 7.76 (d, J = 8.8 Hz, 1H), 7.69 (d, J = 8 Hz, 1H), 7.58 (d, J = 16.4 Hz, 1H), 7.53-7.49 (m, 1H), 7.31-7.27 (m, 1H), 5.54 (s, 2H), 4.61 (t, J = 5.2 Hz, 2H), 4.26 (dd, J = 7.2 Hz, J = 14.4 Hz, 2H), 3.82 (t, J = 5.2 Hz, 2H), 3.47-3.45 (m, 2H), 3.31-3.28 (m, 2H), 3.10 (s, 3H), 1.27 (t, J = 7.2 Hz, 3H). ^{13}C NMR (100 MHz, DMSO-d_6) δ 166.7, 154.5, 145.2, 143.6, 142.0, 140.9, 126.4, 125.3, 126.2, 122.7, 122.1, 121.3, 120.3, 119.9, 119.8, 110.5, 110.3, 71.2, 69.8, 68.8, 14.0. HRMS (MALDI-TOF) m/z Calcd for $C_{28}H_{31}N_2O_4$ 459.2278 Found 459.2296 [M^+].

(*E*)-9-(2-(2-methoxyethoxy)ethyl)-3-(2-(quinolin-4-yl)vinyl)-9H-carbazole (8). To a solution of lepidine (0.8 g, 5.6 mmol) and **5** (1.78 g, 6.0 mmol) in DMF (10 mL) in a sealed tube, chlorotrimethylsilane (6.4 mL, 50 mmol) was added. The resulting mixture was heated to 100 °C for 24 h. After cooling down to 0 °C, water was added and then followed by $NaHCO_3$ aqueous solution to adjust to pH 8. The solution mixture was extracted with dichloromethane three times. The combined organic phase was washed with brine and dried over anhydrous sodium sulfate. After removing the

solvent, the residue was purified by silica gel chromatography affording **8** in 69% yield. ^{1}H NMR (400 MHz, $CDCl_3$) δ 8.87 (d, *J* = 4.4 Hz, 1H), 8.29-8.27 (m, 2H), 8.16-8.13 (m, 2H), 7.82 (d, *J* = 16 Hz, 1H), 7.76-7.70 (m, 2H), 7.60-7.53 (m, 3H), 7.52-7.45 (m, 3H), 7.30-7.26 (m, 1H), 4.50-4.47 (m, 2H), 3.88-3.84 (m, 2H), 3.52-3.50 (m, 2H), 3.44-3.41 (m, 2H), 3.32 (s, 3H). ^{13}C NMR (100 MHz, $CDCl_3$) δ 150.0, 148.8, 143.6, 141.2, 141.1, 136.2, 130.1, 129.4, 128.0, 126.5, 126.4, 124.3, 125.1, 123.7, 123.5, 123.0, 120.6, 119.7, 119.7, 119.6, 116.6, 109.5, 109.3, 72.0, 71.0, 69.4, 59.2, 43.4. HRMS (MALDI-TOF) m/z Calcd for $C_{28}H_{26}N_2O_2$ 423.2083 Found 423.2067 $[M+H]^+$.

(*E*)-1-(2-ethoxy-2-oxoethyl)-4-(2-(9-(2-(2-methoxyethoxy)ethyl)-9H-carbazol-3-yl)vinyl)-quinolin-1-ium bromide (SLAce). A solution of **8** (0.21 g, 0.5 mmol) and ethyl 2-bromoacetate (0.33 g, 2.0 mmol) in ethanol was stirred overnight at room temperature. After removing the solvent, the residue was precipitated from methanol and ethyl acetate affording **SLAce** in 75% yield. ^{1}H NMR (400 MHz, DMSO-*d*$_6$) δ 9.26 (d, *J* = 6.8 Hz, 1H), 9.20 (d, *J* = 8.0 Hz, 1H), 8.90 (d, *J* = 1.2 Hz, 1H), 8.62 (d, *J* = 6.8 Hz, 1H), 8.56 (d, *J* = 15.6 Hz, 1H), 8.45 (d, *J* = 15.6 Hz, 1H), 8.33 (d, *J* = 8.4 Hz, 1H), 8.27-8.23 (m, 2H), 8.16 (dd, *J* = 1.6 Hz, *J* = 8.8 Hz, 1H), 8.06 (t, *J* = 7.2 Hz, 1H), 7.81 (d, *J* = 8.8 Hz, 1H), 7.73 (d, *J* = 8.4 Hz, 1H), 7.55-7.51 (m, 1H), 7.35-7.31 (m, 1H), 6.00 (s, 2H), 4.64 (t, *J* = 5.2 Hz, 2H), 4.23 (dd, *J* = 7.2 Hz, *J* = 14.4 Hz, 2H), 3.86-3.83 (m, 2H), 3.49-3.47 (m, 2H), 3.32-3.31 (m, 2H), 3.11 (s, 3H), 1.27 (t, *J* = 7.2 Hz, 3H). ^{13}C NMR (100 MHz, DMSO-*d*$_6$) δ166.5, 154.6, 148.0, 146.5, 142.4, 141.0, 138.7, 135.4, 129.0, 127.6, 126.7, 126.8, 126.7, 126.5, 126.0, 122.9, 122.2, 122.2, 120.4, 120.0, 118.9, 116.2, 115.0, 110.6, 110.4, 71.3, 69.8, 68.8, 62.3, 58.1, 56.4, 42.9, 14.0. HRMS (MALDI-TOF) m/z Calcd for $C_{32}H_{33}N_2O_4$ 509.2446 Found 509.2427 $[M^+]$.

9-(2-(2-methoxyethoxy)ethyl)-3,6-bis((*E*)-2-(quinolin-4-yl)vinyl)-9H-carbazole (9). To the solution of lepidine (1.6 g, 11.2 mmol) and **6** (1.30 g, 4 mmol) in DMF (10 mL) in a sealed tube, chlorotrimethylsilane (6.4 mL, 50 mmol) was added. The resulting mixture was heated to 100 °C for 24 h. After cooling down to 0 °C, water was added and followed by $NaHCO_3$ aqueous solution to adjust to pH 8. The water solution was extracted with dichloromethane three times. The combined organic phase was washed with brine and dried over anhydrous sodium sulfate. After removing the solvent, the residue was purified by silica gel chromatography affording **9** in 54% yield. ^{1}H NMR

(400 MHz, $CDCl_3$) δ 8.93 (d, J = 4.4 Hz, 2H), 8.41 (d, J = 0.8 Hz, 2H), 8.35 (d, J = 8.0 Hz, 2H), 8.16 (d, J = 8.4 Hz, 2H), 7.92 (d, J = 16 Hz, 2H), 7.81 (dd, J = 1.2 Hz, J = 8.4 Hz, 2H), 7.77-7.73 (m, 2H), 7.68 (d, J = 4.8 Hz, 2H), 7.64-7.60 (m, 3H), 7.56-7.53 (m, 3H), 4.58-4.55 (m, 2H), 3.94 (t, J = 6.0 Hz, 2H), 3.57-3.55 (m, 2H), 3.47-3.45 (m, 2H), 3.33 (s, 3H). ^{13}C NMR (100 MHz, $CDCl_3$) δ 150.5, 149.0, 143.6, 141.7, 136.0, 130.4, 129.5, 128.8, 126.7, 126.6, 125.7, 123.8, 123.6, 120.5, 119.7, 116.8, 110.0, 72.2, 71.2, 69.6, 59.3, 43.8. HRMS (MALDI-TOF) m/z Calcd for $C_{39}H_{33}N_3O_2$ 576.2646 Found 576.2651 $[M+H]^+$.

4,4'-((1*E*,1'*E*)-(9-(2-(2-methoxyethoxy)ethyl)-9H-carbazole-3,6-diyl)bis(ethene-2,1-diyl)) ***bis*****(1-(2-ethoxy-2-oxoethyl)quinolin-1-ium) bromide (VLAce).** A solution of **9** and ethyl 2-bromoacetate in ethanol was stirred overnight at room temperature. After solvent removal, the residue was precipitated from methanol and ethyl acetate affording **VLAce** in 75% yield. ^1H NMR (400 MHz, DMSO-d_6) δ 9.35 (d, J = 8.8 Hz, 2H), 9.30 (d, J = 6.8 Hz, 2H), 9.20 (s, 2H), 8.57 (d, J = 6.8 Hz, 2H), 8.44 (s, 4H), 8.27 (d, J = 8.8 Hz, 2H), 8.20-8.16 (m, 2H), 8.05 (d, J = 8.4 Hz, 2H), 7.99 (t, J = 7.6 Hz, 2H), 7.77 (d, J = 8.8 Hz, 2H), 6.05 (s, 4H), 4.66 (s, 2H), 4.29-4.24 (m, 4H), 3.89-3.87 (m, 2H), 3.53-3.50 (m, 2H), 3.34-3.32 (m, 2H), 3.11 (s, 3H), 1.27 (t, J = 7.2 Hz, 6H). ^{13}C NMR (100 MHz, DMSO-d_6) δ 166.5, 154.4, 147.9, 145.9, 142.9, 138.6, 135.3, 129.0, 127.7, 127.2, 126.0, 123.2, 122.3, 118.8, 116.9, 115.2, 110.9, 71.3, 69.8, 68.9, 62.3, 58.1, 56.4, 14.0. HRMS (MALDI-TOF) m/z Calcd for $C_{47}H_{47}N_3O_6$ 750.3537 Found 750.5489 $[M+1]^+$.

Stability study of the cyanine fluorophores

The photostability of the dyes was studied by spiking the dyes (SPAce, SLAce and VLAce) to 1X Tris Buffer (20 mM Tris-HCl, pH 7.4) with a final concentration of 1 mM, the fluorescence intensity of the dye was recorded every 5 min. To investigate the stability of the dyes against salt, appropriate amount of sodium chloride (NaCl) was spiked into the dye solutions to different final concentrations with and without 100 nM PSA. The fluorescence spectra of the dyes were recorded by a fluorescence spectrophotometer (PTI QM-4/2005).

Coverslips pretreatment and preparation of flow cell

All coverslips are prewashed prior to use. Briefly, No.1 22×22 mm^2 glass slides (Gold Seal, Electron Microscopy System, USA) were sequentially sonicated in household

detergent for 15 min, acetone for 15 min, absolute ethanol for 15 min. Then the coverslips were successively soaked in Piranha (H_2SO_4: H_2O_2 3:1) solution for 30 min, and sonicated for another 30 min. All the coverslips were rinsed with filtered H_2O extensively between each solvent replacement. The slides were finally stored in filtered H_2O and blow-dried with nitrogen gas before use. Sealed flow cell was prepared by combining the prewashed coverslips with double-sided adhesive tape. Each channel was approximately 3-mm in width.

Synthesis of the silica coated iron oxide nanoparticles

Aqueous dispersions of magnetic iron oxide nanoparticles were prepared according to Massart's method.[25] Briefly, 4 mL of 1 M iron (III) chloride hexahydrate (Sigma Aldrich, USA) and 1 mL of 2 M iron (II) chloride tetrahydrate (Sigma Aldrich, USA) in 2 M HCl were added to 50 mL of 0.7 M NH_4OH under rapid mechanical stirring. The black sediment was washed twice with filtered H_2O and re-dispersed in 50 mL filtered H_2O, then three aliquots of 10 mL of 1 M tetramethylammonium hydroxide solution were added to the sediment with rapid mechanical stirring. Finally, the iron oxide nanoparticles were washed with 0.01 M HCl, centrifuged and re-dispersed in filtered H_2O. The silica coating was prepared generally by adding iron oxide nanoparticles to a mixture of ammonium hydroxide, H_2O, and ethanol, then an ethanolic solution of TEOS (Aldrich, USA) (2 mL TEOS in 30 mL ethanol) was added to the mixture under stirring. The final volume of the mixture was 100 mL and the final concentration of the NH_4OH, H_2O, TEOS were 0.35, 21.4 and 0.25 M respectively. The whole hydrolysis and condensation process of the TEOS onto the iron oxide nanoparticles was done in 4 h. The resulted particles were washed with ethanol and H_2O twice and re-suspended in filtered H_2O. The transmission electron microscopy (TEM) imaging of the nanoparticles was done by applying 5 μL of the diluted nanoparticles onto a carbon-coated copper grid (T200H-Cu, Electron Microscopy Science, USA) and dried at ambient condition. Transmission electron micrographs were recorded using a Technai G2 Transmission Electron Microscopy (FEI, USA) with an acceleration voltage of 200 kV.

Preparation of the capture antibody conjugated nanoparticles

Amino-functionalized particles were prepared by using APTES and silylation agent. The functionalization was prepared according to previous reported method.[4] Generally, the APTES was added to the ethanolic solution that contained the silica coated iron oxide nanoparticles (APTES: particles 0.35:1) (w/w) and stirred at 50 °C

for 24 h. The resulting particles were washed twice with ethanol and dispersed in Tris-buffer saline (20 mM Tris-HCl, 50 mM NaCl, pH 7.4) (TBS). Conjugation of the antibody (CHYH1) (Abnova, USA) was done by using the cross-linking reagent, glutaraldehyde (GA). Briefly, the APTES-functionalized particles were added to the TBS solution containing 5 % glutaraldehyde (Sigma-Aldrich) and stirred at room temperature for 1 h. The resultant particles were washed twice with TBS and dispersed in TBS solution. Then 1 nM CHYH1(Ab_1) was incubated with the GA-linked nanoparticles at room temperature for 1 h. The resultant nanoparticles were washed twice with TBS solution, to remove all the excessed CHYH1, and re-dispersed in TBS solution.

Optimization of the performance of the assay

To determine the optimal amount of the detection probe (nanoparticles/ Ab_1 composite), different concentrations of the detection probe, 5, 10, and 20 mg/ml, were incubated with 0 and 50 pM PSA (Sigma-Aldrich, USA) and 200 pM labeling Ab_2, CHYH2 (Abnova, USA), at 37 °C for 1 h. The resultant magnetic immuno-assembly (MIA) was labeled with 100 μM **SLAce** and then injected into the flow cell for fluorescence imaging.

Quantification of cancer antigens with the magnetic immuno-assembly

The calibration curve of the assay was constructed by correlating the average intensity of 50 individual MIA at each concentration of spiked cancer specific antigens, human alpha-fetoprotein (AFP) (Shanghai Linc-bio Science, China), carcinoembryonic antigen (CEA) and PSA of different concentrations (0-20 pM) was incubated with optimal amount of detection probe and 200 pM detection antibodies, anti-human AFP antibody L1C00302 (Shanghai Linc-bio Science, China), anti-human CEA antibody L1C00202 (Shanghai Linc-bio Science, China), CHYH2, respectively, at 37 °C for 1 h. The resultant MIA was labeled with 100 μM **SPAce** (for AFP) **SLAce** (for PSA), and **VLAce** (for CEA). The labeled MIAs were then added to the flow cell channel. The fluorescent images of the MIAs were captured by the home-built total internal reflection fluorescence microscopy (TIRFM) with an excitation 448 nm cyan laser (50 mW, CMA1-01983, Newport, USA).

Quantification of PSA with 10% glycerol

To study the effect of glycerol in the sensitivity of the assay, different concentrations of PSA (0-150 pM) was incubated with optimal amount of detection probe and 200 pM CHYH2 in the presence and absence of 10% glycerol at 37 °C for 1 h. The

resultant MIA was labeled with 100 μM **SLAce**. The **SLAce** labeled MIA were then added to the flow cell channel. The fluorescence images of the MIAs were captured by the TIRFM imaging system.

To study the feasibility of detection with commercial fluorimeter, different concentrations of PSA (0−1 nM) was incubated with optimal amount of detection probe and 5 nM CHYH2 in 10% glycerol-TBS solution at 37 °C for 1 h. The resultant MIA was labeled with 100 μM **SLAce** and the fluorescence spectra of the MIA was recorded by the fluorescence spectrophotometer (PTI QM-4/2005).

Selectivity of the assay

To study the selectivity of the nanoparticles/Ab_1 nanocomposite, four protein samples: Human alpha-fetoprotein (AFP) (Shanghai Linc-bio Science, China), Carcinoembryonic antigen (CEA) (Shanghai Linc-bio Science, China), IgG from rabbit serum (Sigma, USA) and PSA with a final concentration of 10 pM were incubated with optimal amount of CHYH1 conjugated detection probe and 100 pM CHYH2 at 37 °C for 1 h. The MIAs were then labeled with 100 μM **SLAce** and visualized under the TIRFM imaging system.

Enzyme-linked immunosorbent assay (ELISA)

Prostate specific antigen (PSA) ELISA kit was purchased Sigma-Aldrich (USA). The detection of PSA in serum sample was performed as the manufacture's instruction. Briefly, 25 μL of PSA standards and serum sample were added into the streptavidin-coated micro-wells and followed by 100 μL anti-PSA conjugate reagent. Solution mixtures were then incubated at room temperature for 1 hr. The liquid was removed from each well and washed with 300 μL of 1 X wash buffer. Then, 100 μL TMB substrate was added to the wells and incubated at room temperature for 15 min. Finally, 50 μL of stop solution was added into all wells and shaken gently for 15 min. The absorbance at 450 nm was recorded by Benchmark Plus Microplate Reader.

Multiplicity of the assay

To demonstrate the multiplicity of the detection assay 10 pM of AFP, CEA, and PSA were incubated with the optimal amount of detection probe conjugated with the corresponding capturing antibody and labeling antibody at 37 °C for 1 h. For the quantification of AFP, CEA, and PSA in serum, 2 μL of serum with optimal amount of corresponding detection probe and 200 pM detection antibody at 37 °C for 1 h. The immno-assemblies of AFP, CEA and PSA were then labeled with 100 μM SPAce,

VLAce and SLAce respectively and visualized under the TIRFM imaging system. A transmission grating with 70 lines/mm was placed in front of the EMCCD to capture the fluorescence signal from each nano-assembly. The distance between the grating and the EMCCD was set as 23.5 mm, so that the zero and first order image would not overlap.

Imaging system and data analysis

The prism-type total internal reflection fluorescence was setup as mentioned elsewhere.[26,27] Generally, the flow cell was placed between a fused-silica isosceles prism (CVI, laser USA) and a 60 × oil-type objective that equipped on an Olympus IX71 inverted microscope with a BLP-488R long pass filter (Semrock, USA). A 488 nm diode laser (Newport, USA) was used as the excitation source to excite the cyanine fluorophores. The fluorescent image of the nano-assembly was captured by an Electron Multiplying Charge Coupled Device (EMCCD) (PhotonMax 512, Princeton Instrument, USA) incorporated with a Uniphase mechanical shutter (Model LS2Z2, Vincent Associates, USA) and a driver (Model VMM-T1, Vincent Associates, USA) in external synchronization and frame-transfer mode. The exposure time of both camera and shutter were set at 100 ms. The multiplication gain and the delay time of the shutter drive were set at 3000 and 10 ms respectively. In general, fluorescent images of 10 sequential frames each were acquired on different coordinates from a single channel using the WinSpec/32 software (version 2.5.22.0, USA) provided by Princeton Instruments. All the images were analyzed by a public-domain image processing software *Image J.* The fluorescence signals of the MIA were obtained by measuring the net fluorescence intensity of 50 individual MIA randomly. Net average intensity = [(1×1 square pixel on 50 individual MIA) - (1×1 square pixel of 50 independent background area on the image)]/50. All the experiments were done in triplicates and the error bars refer to the standard error of mean of the experiments.

Table S1. Summary of the physical properties of the cyanine fluorophores.

	λ^{abs}_{max} [a] (nm)	λ^{em}_{max} [a] (nm)	Φ_{PL} [b]	Φ_{PL} [c]	λ^{em}_{max} [d] (nm)	$F_{dye\text{-}BSA}/F_{dye}$ [e]
**SPAce**	429	588	0.015	0.20	572	11.2
**SLAce**	478	681	0.0034	0.08	654	15.7
**VLAce**	524	723	0.0007	0.005	707	6.6

[a] measured in phosphate buffer. [b]using Rhodamine 6G (Φ_{488} = 0.95) as standard and measured in PB buffer (pH 7.0). [c]measured in DMSO. [d]Emission maximum upon binding to the PSA in PB buffer (pH 7.0). [e]Fluorescence enhancement ratio upon addition of BSA obtained from fluorescence titration.

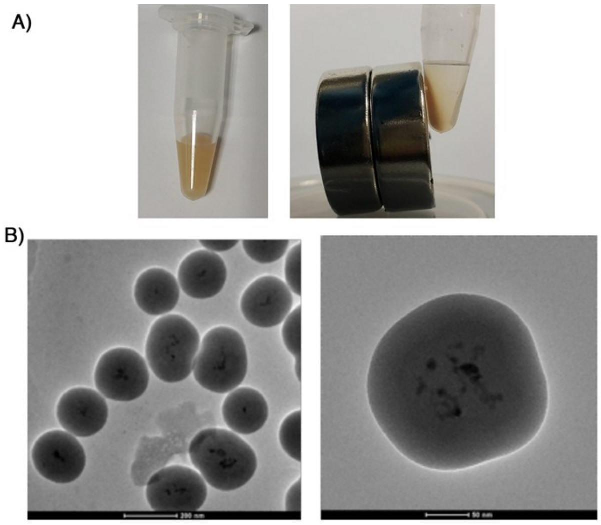

Fig. S1 A) Images of the silica-coated iron oxide nanoparticles in the absence (left) and presence (right) of the external magnetic field. B) TEM image of the silica-coated iron oxide nanoparticle, the average diameter of the particles is 162 ± 25 nm, the average thickness of the silica shell is 55 ± 6 nm and the average diameter of bare iron oxide nanoparticles is 5.3 ± 1.0 nm, n = 50.

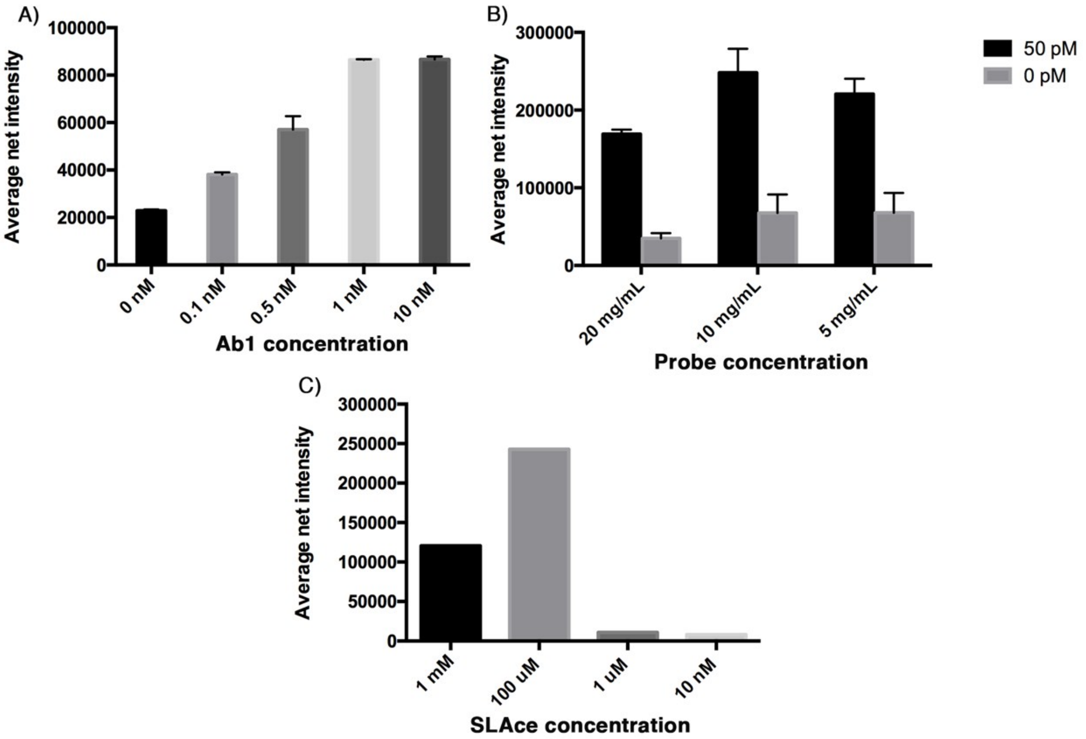

Fig. S5 Optimization of the experimental conditions of the assay. A) Optimizing the applied concentration of the capture antibody that conjugated onto the magnetic nanoparticles, different concentrations of capture antibody CHYH1 (Ab1) were incubated with 10 mg/mL magnetic nanoparticles probe, B) Optimizing magnetic probe concentration, different concentrations (20, 10, and 5 mg/mL) of the magnetic nanoparticle probes were used in the presence of 0 and 50 pM PSA, C) Optimizing the labelling dye concentration, different concentrations of **SLAce** were added to label immuno-assemblies having 10 pM PSA. Error bar, standard error of mean, n = 3. Net average intensity = [50×(1×1 square pixel on the MIA)- 50×(1×1 square pixel of independent background are on the image)]/ 50.

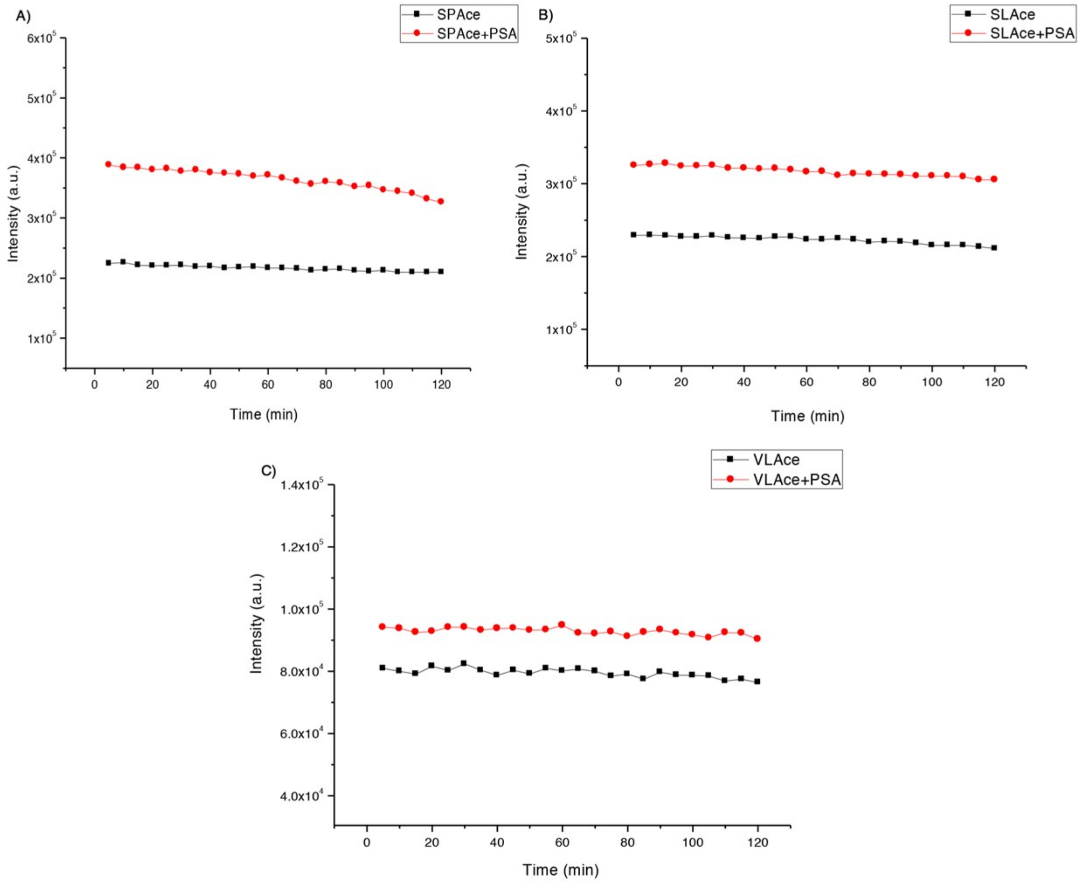

Fig. S3 Photostability study of A) **SPAce**, B) **SLAce** and C) **VLAce** before and after binding to PSA in PB buffer (pH 7.0).

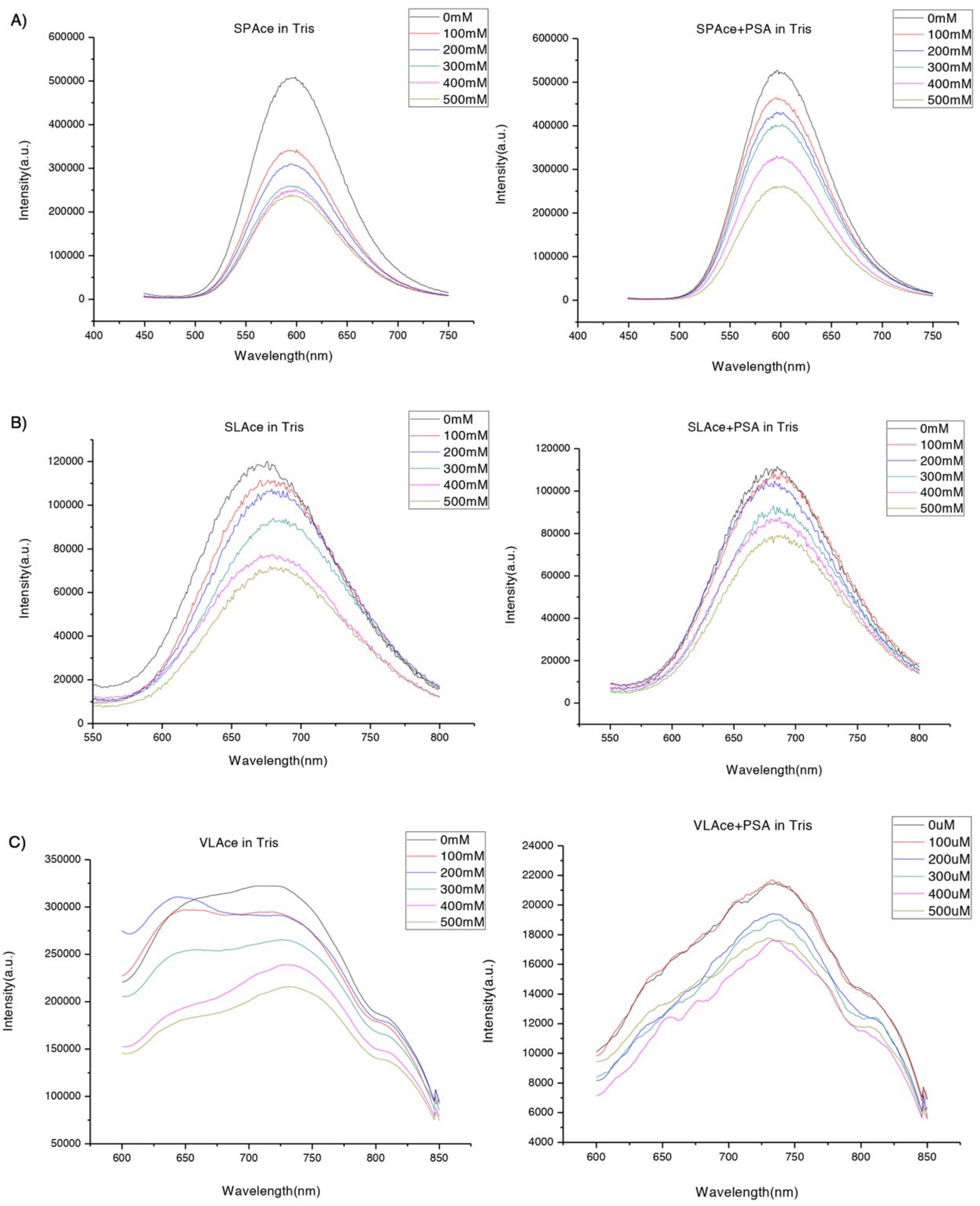

Fig. S4 The fluorescence response of A) **SPAce**, B) **SLAce**, and C) **VLAce** against salt concentration (NaCl) in the absence (left) or presence (right) of PSA in 1X Tris Buffer (20 mM Tris-HCL, pH 7.4).

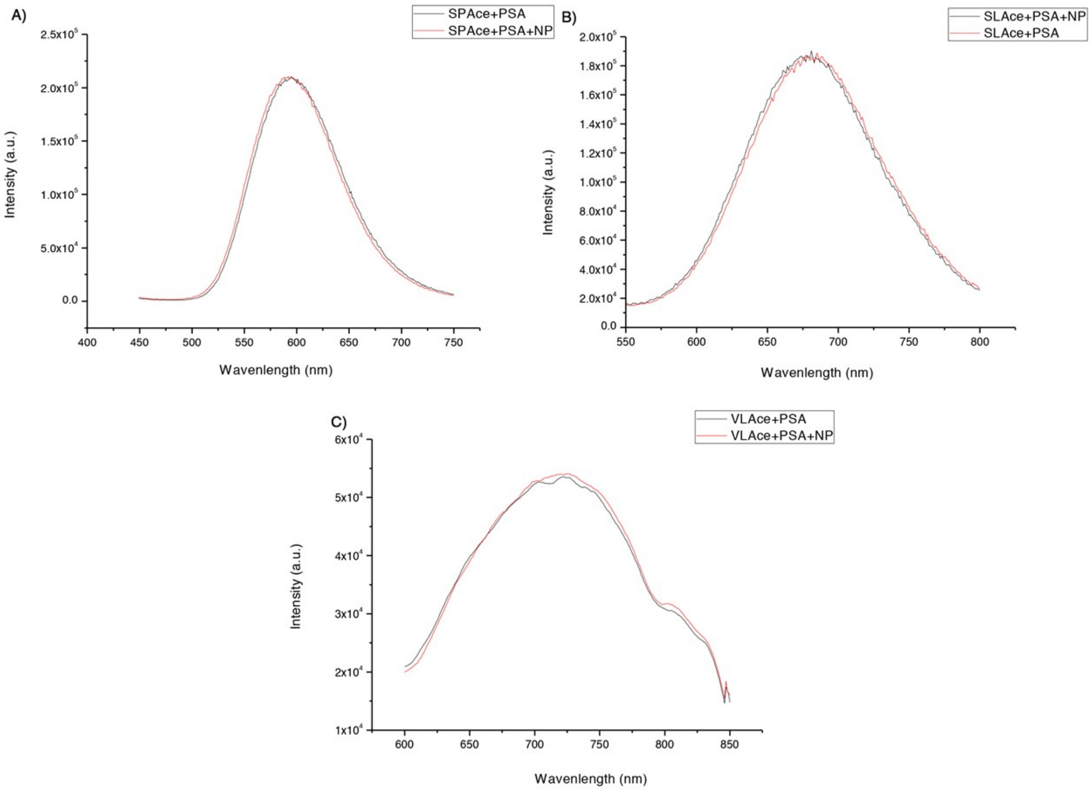

Fig. S5 The influence of the magnetic nanoparticles on the fluorescence response of the three cyanines upon binding to the protein.

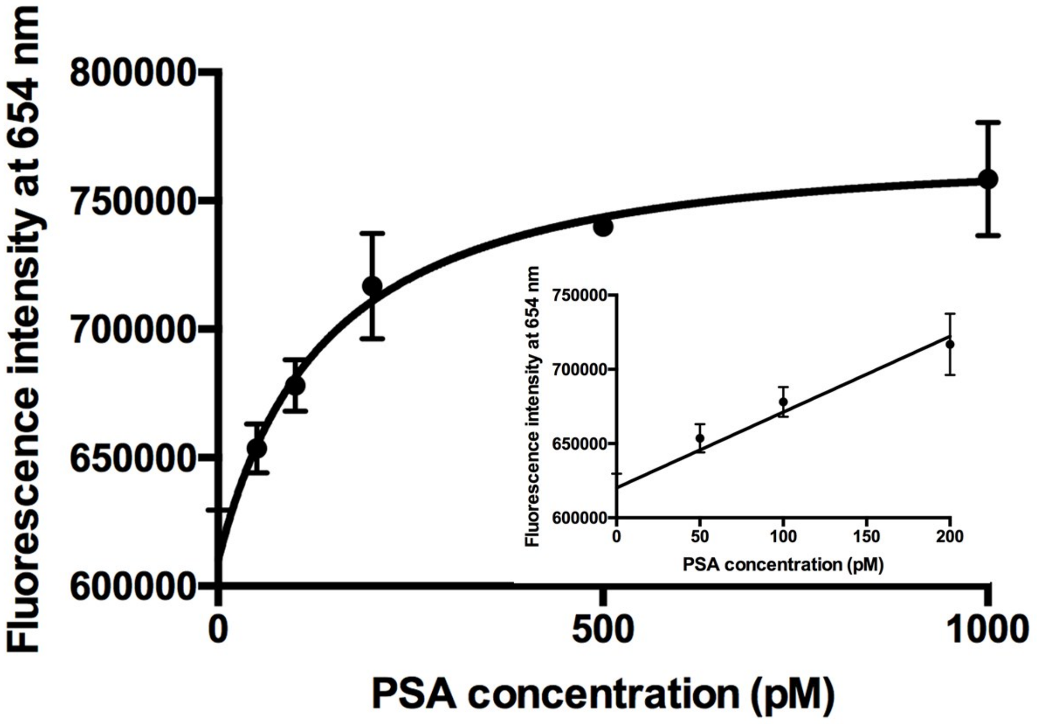

Fig. S6 The quantification PSA labeled with **SLAce** in the presence of 10% glycerol measured by a spectrofluorimeter. A linear range of 0−200 pM of PSA was obtained.

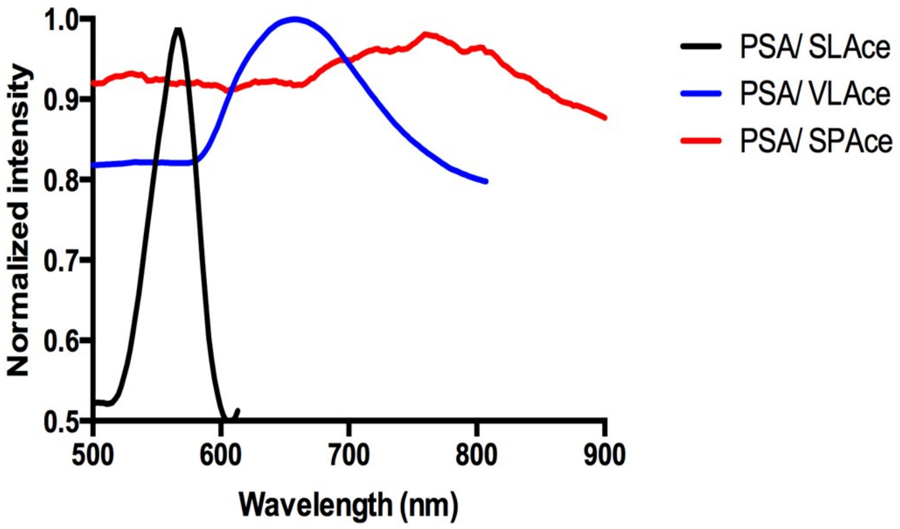

Fig. S7 Emission spectra, obtained from the TIRFM-grating system, of the PSA magnetic immune-assemblies upon binding to **SPAce**, **SLAce**, and **VLAce**, respectively.

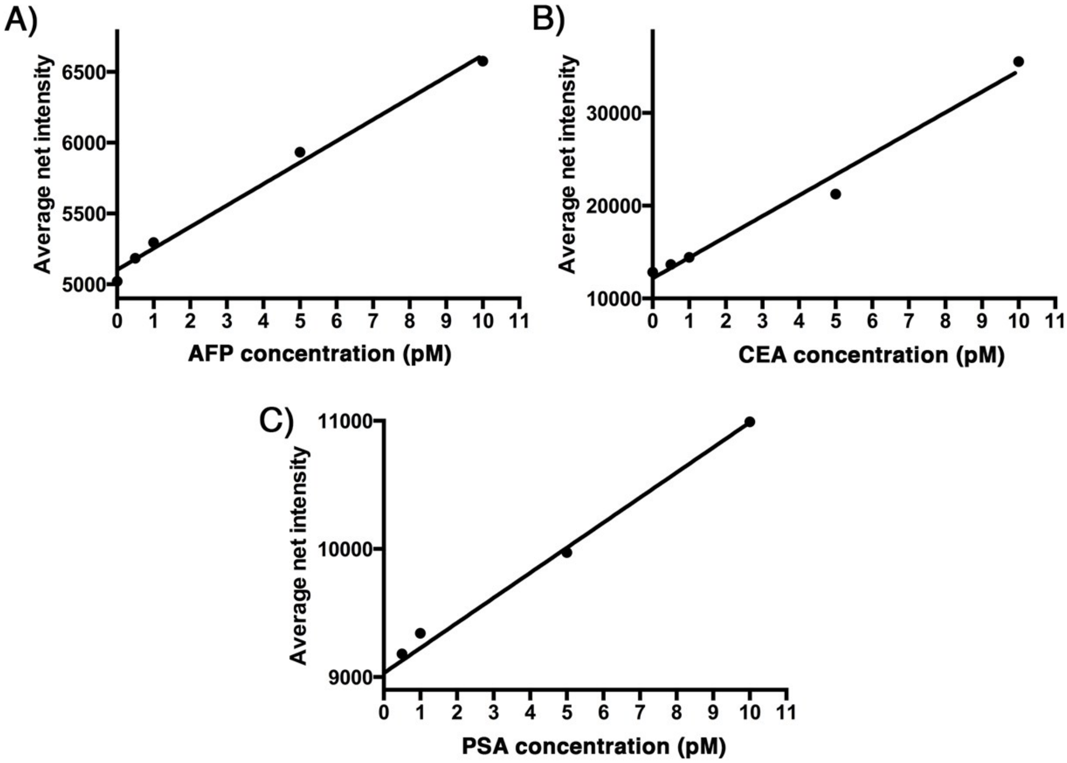

Fig. S8 Standard curves for quantification multiplex quantification of cancer associated antigens: A) AFP, B) CEA, and C) PSA. Error bar, standard error of mean, n = 3. (Average net intensity = (1×1 sq pixel of 50 individual MIAs) – (1×1 sq pixel of 50 individual background area on the image)/50).

References:

1. X. J. Feng, P. Z. Tian, Z. Xu, S. F. Chen, M. S. Wong, *J. Org. Chem.* 2013, **78,** 11318-11325.
2. X. J. Feng, P. L. Wu, F. Bolze, H. W. C. Leung, K. F. Li, N. K. Mak, D. W. J. Kwong, J.-F. Nicoud, K. W. Cheah, M. S. Wong, *Org. Lett.* 2010, **12,** 2194-2197.
3. W. Yang, Y. Wong, O. T. W. Ng, L.-P. Bai, D. W. J. Kwong, Y. Ke, Z.-H. Jiang, H.-W. Li, K. K. L. Yung and M. S. Wong, *Angewandte Chemie-International Edition*, 2012, **51**, 1804-1810.
4. S. A. Fiel, H. Yang, P. Schaffer, S. Weng, J. A. H. Inkster, M. C. K. Wong and P. C. H. Li, *ACS Applied Materials & Interfaces*, 2015, **7**, 12923-12929.
